# Gait Speed and Mortality Risk Among Community-Dwelling Older Adults with Heart Disease: Evidence from the RAND HRS

**DOI:** 10.1093/geroni/igaf122.3596

**Published:** 2025-12-31

**Authors:** Melanie Navos, Yong-Fang Kuo

**Affiliations:** University of Texas Medical Branch, Galveston, Texas, United States; University of Texas Medical Branch, Galveston, Galveston, Texas, United States

## Abstract

We examined the relationship of heart disease and gait speed with all-cause mortality over 11 years of follow-up among community-dwelling older adults. Participants (N = 1153) were from the RAND Health and Retirement Study Longitudinal 2020 (v2) dataset, a nationally-representative longitudinal study that began in 1992. Measures included socio-demographics, multi-morbidity status, depressive symptoms, cognitive function, and all-cause mortality. We used Cox-Proportional Hazards to estimate the hazard ratio (HR) and 95% confidence interval (CI) of all-cause mortality as a function of gait speed. We also examined the nonlinear form of gait speed to obtain the best cut-off points for predicting mortality that are more applicable in the clinical setting than its linear form. Participants were categorized into four groups based on gait speed cut-off (≤0.5 m/s, 0.5-≤0.75 m/s, 0.75-≤1.0 m/s, >1m/s). Older adults with heart disease and slower gait speed of ≤ 0.5m/sec are more at higher risk for mortality (HR = 3.02, 95% CI = 2.18-3.06), while, those with gait speed 0.5-≤0.75 m/s are at moderately elevated risk for mortality (HR = 2.25, 95% CI 1.61-3.16), and those with gait speed 0.75-≤1.0 m/s are at lower risk than the slow walking groups (HR = 1.85, 95% CI 1.38-2.47), compared to those with gait speed >1m/sec after controlling for all covariates. These findings underscore the value of gait speed as a prognostic marker and highlight the need for targeted interventions such as lower-extremity strengthening and endurance training to preserve mobility and reduce mortality risk in this vulnerable population.

